# Is Glaucoma a Neurodegeneration caused by Central Insulin Resistance: Diabetes Type 4?

**DOI:** 10.5005/jp-journals-10028-1228

**Published:** 2017-10-27

**Authors:** Tanuj Dada

**Affiliations:** Professor, Dr Rajendra Prasad Centre for Ophthalmic Sciences, All India Institute of Medical Sciences, New Delhi, India

## Abstract

**How to cite this article:** Dada T. Is Glaucoma a Neurodegeneration caused by Central Insulin Resistance: Diabetes Type 4? J Curr Glaucoma Pract 2017;11(3):77-79.

## INTRODUCTION

Glaucoma is an acquired multifactorial progressive neurodegenerative syndrome with complex pathogenesis. It is characterized by accelerated retinal ganglion cell (RGC) apoptosis and leads to optic neuropathy with visual field defects, the intraocular pressure (IOP) being a major risk factor. The only therapy currently available is lowering of IOP with medical/laser/surgical therapy; however, many patients continue to progress despite an adequately controlled or apparently “normal” IOP. Recent studies have shown that glaucoma patients have central neurodegenera-tion involving both the occipital cortex and lateral genicu-late body (LGB).^1,2^ It has been postulated that glaucoma may be a disease initially affecting the central nervous system (CNS) and traveling downstream to the optic nerve and RGCs. Our group recently proposed a novel theory for glaucoma—the brain diabetes theory (describing glaucoma as diabetes type 4).^3,4^ This theory explains that glaucoma is a CNS condition involving brain insulin resistance or central insulin signaling dysfunction, which leads to transsynaptic neurodegeneration. This proposal has given rise to a new holistic theory for primary open angle glaucoma (POAG)/normal pressure glaucoma and raised the possibility of the development of therapeutic approaches targeting the brain rather than the eye.

Diabetes types I and II are insulin hypofunctionality-mediated systemic syndromes accompanied by hypo-insulinemia (type I) or insulin resistance (type II). But the existence of brain-specific diabetes independent of peripheral diabetes and manifesting as neurodegenera-tion has been previously reported as diabetes type III— Alzheimer’s disease (AD)^5^ and recently as diabetes type 4—glaucoma.^4^

## THE ROLE OF INSULIN IN THE BRAIN AND THE EYE

Insulin/insulin signaling is important for neuronal survival, in general,^6^ and RGC survival, in particular.^7^ Insulin is present in the brain in very high (10-100 times the level in plasma) quantities,^8^ indicating local production and its important role in cerebral functions. Additionally, insulin is important in trabecular meshwork (TM) maintenance and aqueous outflow regulation, thereby playing a role in maintenance of IOP. Ameliorating defects in insulin signaling is, therefore, an important therapeutic target to consider for glaucoma therapy.

- Insulin has been found to be important in the production of nitric oxide (NO) by TM cells mediated through *de novo* synthesis of tetrahydrobiopterin. The NO is important in aqueous outflow regulation and has been reported to increase outflow^9-11^ and, hence, decrease the IOP. This means insulin resistance may cause elevation in IOP leading to ocular hypertension and POAG, and insulin-based therapy may have role to play in lowering IOP through enhancement of aqueous outflow.
- Mitochondrial dysfunction leading to oxidative stress lies at the center stage of glaucomatous damage and insulin is required for healthy functioning of the mitochondria. An increase in IOP leads to mitochondrial dysfunction,^12^ and this, in turn, leads to aberrant insulin signaling, which creates a vicious self-perpetuating cycle^13^ with serious damage to mitochondrial functions and an increase in oxidative injury to RGCs. Elevated IOP also leads to mitochondrial fission and optic nerve head cupping mediated by release of OPA1, an important gene involved in various forms of optic neuropathy^14^ including optic nerve head atrophy.^15^
- Insulin induces the expression of GLUT4 in the RGC layer of retina,^16^ thereby enhancing RGC survival. Insulin deprivation or resistance can, therefore, lead to impaired RGC function and trigger apoptosis and cell death. This can occur independent of any increase in IOP. Since RGC layer of retina is metaboli-cally highly active, its activity and cellular viability depend on a continuous uninterrupted supply and uptake of glucose. This constant supply of energy of this energetically expensive layer is ensured by insulin-mediated expression of GLUT4.
- Insulin is required for neuronal survival^6,7^ and functions as an antiapoptotic hormone,^17^ present in the brain in far higher quantities than in the systemic circulation. Insulin restores metabolic function in neurons under oxidative stress.
- Insulin is also an anti-inflammatory moiety^18^ with an important role to play in preventing glial activation as insulin resistance is associated with gliosis.^19^ Glial activation (astrocytes/microglia) is one of the earliest events in glaucoma pathogenesis. This indicates that insulin signaling derangement is one of the earliest events in glaucoma pathogenesis and this signaling pathway can serve as an early therapeutic target.
- Glutamate excitotoxicity has been implicated in glaucoma pathogenesis in several studies. Insulin has been found to be neuroprotective and observed to prevent toxicity in glutamate-induced excitotoxic conditions.^20^ Also, glutamate excitotoxicity in neuronal cell lines leads to elevation in reactive oxygen species (ROS),^21,22^ and this elevation in ROS can be brought down by increasing insulin concentration in these cell lines.^21,22^
- Impairment of insulin signaling leads to increased phosphorylation of microtubule-associated protein Tau, predominantly found in axons, a hallmark of neurodegenerative disease. The presence of abnormal tau protein can impair axonal growth and viability and this protein can be found in optic nerve head and RGCs in glaucoma.^23^
- Insulin resistance leads to accumulation of amyloid beta (Ap) in the CNS (Alzheimer’s) and ocular tissues (RGC, occipital cortex). Increased deposition of Ap-plaques has been observed in retinas of glaucoma patients^24^ and associated with an increase in RGC apoptosis.^25-28^ Another pathological change observed in human visual cortex due to accumulation of amyloid is cerebral amyloid angiopathy leading to vascular dysregulation and ischemia. Enhancing insulin function may help in preventing plaque deposition and preserving RGC function.

**Fig. 1:**
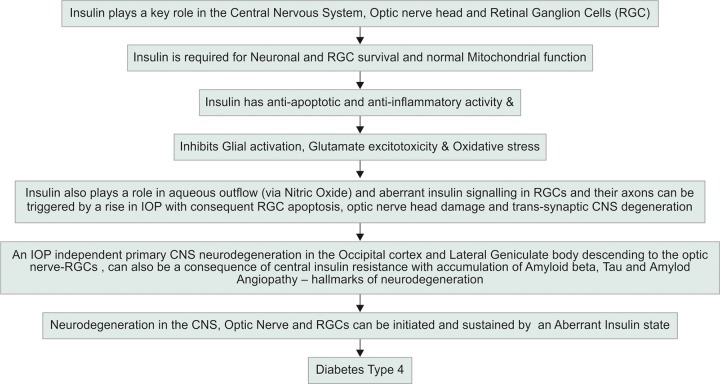
Role of insulin resistance in glaucoma

In summary, the central insulin resistance theory (Fig. 1) explains how insulin dysfunction can specifically cause both forms of glaucoma (high pressure and normal pressure) by afflicting outflow pathways via TM, vascular changes (amyloid angiopathy), and trigger glial activation, central neuronal degeneration, and RGC apoptosis through various molecular pathways. Therefore, aberrant insulin signaling in the CNS and specifically visual pathways (RGC-optic nerve-LGB-occipital cortex) appears to be a cause for glaucoma. Central insulin functional enhancement by giving intranasal insulin therapy may help to lower IOP, enhance blood flow, and ameliorate injury to RGCs, preventing RGC apoptosis by positively modulating several cellular pathways like glial activation, glutamate excitotoxicity, ameliorating amyloidopathy/ taupathy, and decreasing mitochondrial dysfunction.
